# Supplementary material for: Confounding factors in algal phosphorus limitation experiments
Source: PLoS One. 2018 Oct 18;13(10):e0205684. doi: 10.1371/journal.pone.0205684 (PMC6193650; doi:10.1371/journal.pone.0205684)
Supplement: S1 Table — Multiple measurements are recorded as mean ± standard deviation. Several nutrient measurements were below detection. pH was not measured during experiment 3 because of an instrument malfunction. (DOCX) [file pone.0205684.s001.docx]

**S1 Table.** **Environmental variables measured during nutrient diffusing substrate experiments at Little Beaver Creek, CO.** Multiple measurements are recorded as mean ± standard deviation. Several nutrient measurements were below detection. pH was not measured during experiment 3 because of an instrument malfunction.
