# Supplementary material for: Confounding factors in algal phosphorus limitation experiments
Source: PLoS One. 2018 Oct 18;13(10):e0205684. doi: 10.1371/journal.pone.0205684 (PMC6193650; doi:10.1371/journal.pone.0205684)
Supplement: S2 Table — Four different phosphate chemicals (KH2PO4, K2HPO4, NaH2PO4, Na2HPO4) crossed with laboratory heating methodology were deployed in two NDS experiments in 2016. Treatment classes in the statistical analyses included phosphate cation, phosphate form, and heating method. ANOVA results testing the effect of each treatment class on each response variable’s P treatment effect size are presented, with experiment included as a fixed effect block in all models. P<0.05 is indicated as “*”. (DOCX) [file pone.0205684.s002.docx]

**S2 Table**. **Results of ANOVAs testing the effect of three treatment classes on P effect sizes.** Four different phosphate chemicals (KH_2_PO_4_, K_2_HPO_4_, NaH_2_PO_4_, Na_2_HPO_4_) crossed with laboratory heating methodology were deployed in two NDS experiments in 2016. Treatment classes in the statistical analyses included phosphate cation, phosphate form, and heating method. ANOVA results testing the effect of each treatment class on each response variable’s P treatment effect size are presented, with experiment included as a fixed effect block in all models. P<0.05 is indicated as “*”.

|  | Chl *a* | | AFDM | | AI | | GPP | | GPP/Chla | |
| --- | --- | --- | --- | --- | --- | --- | --- | --- | --- | --- |
| Factor | *F* | *p* | *F* | *p* | *F* | *p* | *F* | *p* | *F* | *p* |
| Cation | 0.341 | 0.561 | 0.352 | 0.555 | 0.407 | 0.526 | 0.167 | 0.684 | 0.632 | 0.429 |
| Expt | 0.059 | 0.809 | 0.665 | 0.417 | 0.887 | 0.349 | 6.368 | 0.013* | 6.385 | 0.013* |
| Form | 0.571 | 0.452 | 1.552 | 0.217 | 2.262 | 0.137 | 5.057 | 0.027* | 5.578 | 0.020* |
| Expt | 0.080 | 0.778 | 0.673 | 0.415 | 0.913 | 0.342 | 6.417 | 0.013* | 6.448 | 0.013* |
| Heat | 0.498 | 0.482 | 0.262 | 0.610 | 0.528 | 0.470 | 0.351 | 0.555 | 0.011 | 0.917 |
| Expt | 0.080 | 0.779 | 0.591 | 0.444 | 0.789 | 0.377 | 6.449 | 0.013* | 6.589 | 0.012* |
