# Supplementary material for: Confounding factors in algal phosphorus limitation experiments
Source: PLoS One. 2018 Oct 18;13(10):e0205684. doi: 10.1371/journal.pone.0205684 (PMC6193650; doi:10.1371/journal.pone.0205684)
Supplement: S3 Table — Four different phosphate chemicals (KH2PO4, K2HPO4, NaH2PO4, Na2HPO4) crossed with laboratory heating methodology and two phosphate concentrations were deployed in an NDS experiment in 2017. Treatment classes in the statistical analyses included phosphate cation, phosphate form, and heating method, along with concentration as a potential modifier. Two-way ANOVA results are presented where each treatment class was crossed with concentration, to determine effects on each response variable’s P treatment effect size. P<0.05 is indicated as “*”. (DOCX) [file pone.0205684.s003.docx]

**S3 Table.** **Results of ANOVAs testing the effect of four treatment classes on P effect sizes.** Four different phosphate chemicals (KH_2_PO_4_, K_2_HPO_4_, NaH_2_PO_4_, Na_2_HPO_4_) crossed with laboratory heating methodology and two phosphate concentrations were deployed in an NDS experiment in 2017. Treatment classes in the statistical analyses included phosphate cation, phosphate form, and heating method, along with concentration as a potential modifier. Two-way ANOVA results are presented where each treatment class was crossed with concentration, to determine effects on each response variable’s P treatment effect size. P<0.05 is indicated as “*”.

|  | Chla | | AFDM | | | AI | | GPP | | GPP/Chla | |
| --- | --- | --- | --- | --- | --- | --- | --- | --- | --- | --- | --- |
| Factor | *F* | *p* | | *F* | *p* | *F* | *p* | *F* | *p* | *F* | *p* |
| Cation | 0.124 | 0.725 | | 0.275 | 0.601 | 0.079 | 0.800 | 0.044 | 0.835 | 0.234 | 0.630 |
| Conc | 0.263 | 0.609 | | 1.231 | 0.270 | 0.901 | 0.345 | 0.767 | 0.384 | 0.107 | 0.744 |
| Cation*Conc | 1.507 | 0.223 | | 0.060 | 0.806 | 0.311 | 0.578 | 0.858 | 0.357 | 0.000 | 1.000 |
| Form | 0.124 | 0.725 | | 4.129 | 0.450 | 3.636 | 0.060 | 2.850 | 0.095 | 2.091 | 0.152 |
| Conc | 0.291 | 0.591 | | 1.239 | 0.269 | 0.828 | 0.365 | 0.817 | 0.369 | 0.105 | 0.747 |
| Form*Conc | 0.586 | 0.446 | | 0.097 | 0.756 | 0.008 | 0.927 | 0.475 | 0.493 | 1.045 | 0.309 |
| Heat | 0.700 | 0.405 | | 0.005 | 0.944 | 0.296 | 0.588 | 2.234 | 0.138 | 5.540 | 0.021* |
| Conc | 0.359 | 0.550 | | 1.233 | 0.270 | 0.790 | 0.376 | 0.690 | 0.408 | 0.050 | 0.823 |
| Heat*Conc | 0.516 | 0.474 | | 1.199 | 0.276 | 2.875 | 0.093 | 1.159 | 0.284 | 1.156 | 0.285 |
